# Analysis of Lipophilic Antioxidants in the Leaves of *Kaempferia parviflora* Wall. Ex Baker Using LC–MRM–MS and GC–FID/MS

**DOI:** 10.3390/antiox10101573

**Published:** 2021-10-05

**Authors:** Kihwan Song, Ramesh Kumar Saini, Young-Soo Keum, Iyyakkannu Sivanesan

**Affiliations:** 1 Department of Bioresource Engineering, Sejong University, 209 Neungdong-ro, Gwangjin-gu, Seoul 05006, Korea; khsong@sejong.ac.kr; 2 Department of Crop Science, Konkuk University, Seoul 05029, Korea; saini_1997@yahoo.com (R.K.S.); rational@konkuk.ac.kr (Y.-S.K.); 3 Department of Bioresources and Food Science, Institute of Natural Science and Agriculture, Konkuk University, Seoul 05029, Korea

**Keywords:** black ginger, byproducts, carotenoids, fatty acids, phylloquinone, tocopherols, phytosterols

## Abstract

Lipophilic metabolites such as carotenoids, fatty acids, vitamin K1, phytosterols, and tocopherols are important antioxidants that are used in the cosmetics, foods, and nutraceutical industries. Recently, there has been a growing demand for the use of byproducts (wastes) as a potential source of industrially important compounds. The leaves of *Kaempferia parviflora* (black ginger) (KP-BG) are major byproducts of KP-BG cultivation and have been reported to contain several bioactive metabolites; however, the composition of lipophilic metabolites in KP-BG leaves has not been examined. In this study, the lipophilic antioxidant profile was analyzed in the leaves of KP-BG plants grown in vitro and ex vitro. Lipophilic compounds, namely carotenoids (80.40–93.84 µg/g fresh weight (FW)), tocopherols (42.23–46.22 µg/g FW), phytosterols (37.69–44.40 µg/g FW), and vitamin K1 (7.25–7.31 µg/g FW), were quantified using LC–MRM–MS. The fatty acid profile of the KP-BG leaves was identified using GC–FID/MS. The content of individual lipophilic compounds varied among the KP-BG leaves. Ex vitro KP-BG leaves had high levels of lutein (44.38 µg/g FW), α-carotene (14.79 µg/g FW), neoxanthin (12.30 µg/g FW), β-carotene (11.33 µg/g FW), violaxanthin (11.03 µg/g FW), α-tocopherol (39.70 µg/g FW), α-linolenic acid (43.12%), palmitic acid (23.78%), oleic acid (12.28%), palmitoleic acid (3.64%), total carotenoids (93.84 µg/g FW), and tocopherols (46.22 µg/g FW) compared with in vitro KP-BG leaves. These results indicate that ex-vitro-grown KP-BG leaves could be used as a valuable natural source for extracting important lipophilic antioxidants.

## 1. Introduction

The genus *Kaempferia* Linn. (Zingiberaceae) includes approximately 60 medicinal herbs native to Southeast Asia, China, and India [1]. *Kaempferia* species have been used in indigenous medicine for centuries [2]. *Kaempferia* extracts possess antimicrobial [3], antinociceptive [4], antitumor [5], antiallergenic [6], antiobesity [7], antiviral [8], antioxidant [9], anticholinesterase [10], anti-inflammatory [11], neuroprotective [12], and wound healing [13] properties. Several bioactive metabolites, namely diterpenoids, flavonoids, phenolics, steroids, triterpenes, and volatile oils, have been identified in *Kaempferia* species (reviewed in [2]).

*Kaempferia parviflora,* known as black ginger (KP-BG), is distributed in India, Laos, Myanmar, and Thailand. KP-BG is commercially produced in Thailand and other Southeast Asian countries using rhizomes. The rhizome of KP-BG is used to treat infantile colic, weakness, diabetes, male impotence, gout, and ulcers in folk medicine. Methoxyflavones (5,7-dimethoxyflavone, 3,4,5,7-tetramethoxyflavone, 5-hydroxy-3,7,3,4-tetramethoxyflavone, 5,3′-dihydroxy-3,7,4′-trimethoxyflavone, 5,7,4′-trimethoxyflavone, and 3,5,7,4,5-pentamethoxyflavone), kaempferiaosides, and terpenoids obtained from KP-BG rhizome extracts have been reported to possess anti-allergic [6], adaptogenic [14], antimutagenic [15], hepatoprotective [16], anti-osteoporotic [17], and antioxidant [17] properties. The foliage of *Kaempferia* spp., a major byproduct of *Kaempferia* cultivation, is also considered a good source of bioactive metabolites [18,19,20,21,22,23], and has been reported to have antinociceptive, anti-inflammatory [20], sedative [21], antioxidant, and tyrosinase inhibition [22] properties. The leaves of *Kaempferia galanga* and *Kaempferia rotunda* are also used as food flavoring agents and for preparing beverages [22,24,25]. The total carotenoid, flavonoid, phenolic, essential oil, and alpha-tocopherol contents in leaves of several *Kaempferia* spp. have been reported [22,23,26,27]. Chan et al. [22] reported that the total phenolic content and antioxidant capacity were higher in the leaf extract than in the rhizome extract of *K. galanga*. To date, there is only one study [23] on the bioactive metabolite profile and biological activity of KP-BG foliage extract. Park et al. [23] screened bioactive metabolites in both in-vitro-cultured and ex-vitro-grown KP-BG plant leaves. Their results showed the presence of many bioactive metabolites (phenolics and flavonoids) in KP-BG leaves that were previously reported in rhizomes. However, no literature is available about the lipophilic compounds present in the leaves of KP-BG. Carotenoids, fatty acids, phylloquinone, phytosterols, and tocopherols are lipophilic compounds that play important roles in plant development and defense against stress [28,29,30,31]. These lipophilic nutrients are also crucial for human health [32,33] because of their antioxidant, antiaging, anti-inflammatory, anticancer, and cardioprotective properties [34,35]. Thus, there is a growing demand for the evaluation of lipophilic substances in plants. The content of bioactive metabolites in field-grown plants is often influenced by climatic and soil conditions. In the present study, the profile and content of lipophilic compounds in KP-BG leaves of in-vitro-raised plantlets and ex-vitro (polyhouse)-grown plants were investigated. The analysis and quantification of these antioxidant components using LC–MRM–MS and GC–FID/MS is documented for the first time. The results show the presence of five classes of metabolites, namely carotenoids, fatty acids, phylloquinone, phytosterols, and tocopherols, in KP-BG byproducts that have beneficial effects on human health. The levels of several lipophilic compounds were higher in ex vitro KP-BG leaves than the in vitro KP-BG leaves. The high content of lipophilic metabolites, mainly α-carotene in KP-BG leaves, encourages scientists to explore this plant further.

## 2. Materials and Methods

### 2.1. Standards and Chemicals

Authentic standards of phylloquinone (Vitamin K1), γ-tocopherol (γ-Toc), β-tocopherol (β-toc), α-tocopherol (α-Toc), 24α-ethyl cholesterol, 24α-methyl cholesterol, trans-β-apo-8′-carotenal (internal standard), 5-α-Cholestan-3β-ol (internal standard), 37-component fatty acid methyl esters (FAME) mix, CRM47885 (standard fatty acid), ammonium formate (NH_4_HCO_2_), boron trifluoride (BF_3_)–methanol (MeOH) solution (14% in methanol), butylated hydroxytoluene (Bht), cyclohexane (Cyhex), dichloromethane (Dcm), isopropyl alcohol (Ipa), magnesium carbonate (MgCO_3_), sodium sulfate anhydrous (Na_2_SO_4_), sodium chloride (NaCl), and magnesium oxide (MgO) were purchased from Merck Ltd., Seoul, Korea. Acetone (Ace), water (H_2_O), hexane (Hex), methyl tert-butyl ether (MTBE), methanol (MeOH), chloroform (CHCl_3_), and ethanol (EtOH) were acquired from Daejung, Siheung-si, Korea. The major carotenoids, namely (all-*E*)-violaxanthin (Vio), (all-*Z*)-neoxanthin (Neo), (all-*E*)-lutein (Lut), and (all-*E*)-β-carotene (β-Car), used in this study were purified from lettuce (Romaine green: *Lactuca sativa* var. *longifolia* ‘Esse’) using preparative thin-layer chromatography (TLC), employing a kieselguhr and magnesium oxide (1:1, *w/w*)-coated TLC plate, and a mobile phase of Ace: Hex (1:1, *v/v*) [36]. Similarly, another carotenoid, (all-*E*)-α-carotene (α-Car), was purified from carrots.

### 2.2. Plant Material

Leaves (3 months old) of KP-BG were collected from in-vitro-raised plantlets and ex-vitro (polyhouse)-grown plants. KP-BG plantlets were raised according to Park et al. [23]. Briefly, surface-sterilized rhizome buds of KP-BG were cultivated on Murashige and Skoog (MS) basal medium with agar (0.8%), sucrose (3%), 6-benzyladenine (8.0 µM), and thidiazuron (0.5 µM) under 24 ± 1 °C and a 16 h photoperiod (40 µmol s^−1^ m^−2^) for shoot induction. After four weeks, the regenerated KP-BG shoots were grown on medium MS with agar (0.8%), sucrose (3%), and indole-3-butyric acid (2.0 µM) under 24 ± 1°C and a 16 h photoperiod (45 µmol s^−1^ m^−2^) for root induction. The cultures were subcultured on the same fresh rooting medium at 6-week intervals. After 6 weeks, rooted KP-BG plantlets were planted into plastic trays containing a mixture of peat moss, perlite, and vermiculite (40:30:30) and maintained in a growth room under a 16 h photoperiod (90 µmol s^−1^ m^−2^) at 24 ± 1 °C. They were fertigated with MS basal nutrient solutions at three-day intervals. After 5 weeks, well-developed KP-BG plantlets were transplanted into plastic pots containing peat moss, perlite, and vermiculite (40:30:30) and maintained in a polyhouse under a 12 h photoperiod (230 µmol s^−1^ m^−2^) at 26 ± 1 °C. They were fertigated with Hyponex nutrient solution (2 g L^−1^; N:P:K; 20:20:20) every four days. Lettuce and carrot were procured from E-mart (Seoul, Korea).

### 2.3. Analysis of Lipophilic Metabolites Using Liquid Chromatography–Multiple Reaction Monitoring–Mass Spectrometry (LC–MRM–MS)

The lipophilic metabolites were extracted from KP-BG leaves using Ace:Hex:EtOH (1:2:1) containing 0.1% (*w/v*) Bht (lipophilic extraction (LE) solution) as previously described [37]. Briefly, 4 g (exact to 0.001 g) of KP-BG leaves was placed in an amber glass vial containing 40 µL of trans-β-apo-8′-carotenal and 5-α-Cholestan-3β-ol (internal standards, dissolved in Ace, 1 mg/mL), a pinch of MgCO_3_, and 10 mL of LE solution. Samples were homogenized, sonicated (300 W, 60 Hz) for 15 min (JAC-2010, Sonics & Materials Inc., Newtown, CT, USA), centrifuged (5000× *g* for 5 min at 4 °C), and the supernatant was collected. The sample was re-extracted with 10 mL of fresh LE solution until the extract became colorless. Combined supernatants were evaporated at <35 °C using a rotary vacuum evaporator (RE 111, Büchi, Flawil, Switzerland), reconstituted with 4 mL of Ace comprising 0.1% BHT, and filtered (0.45 µm, Nylon syringe filter, Whatman) into an amber vial for LC–MRM–MS analysis.

Conditions for quantitative lipophilic antioxidant determination were adopted as described by Kim et al. [38]. Analysis was performed on a triple quadrupole mass spectrometer (SCIEX API 3200™, Applied Biosystems/MDS SCIEX, CA, USA) equipped with liquid chromatography (Exion LC™ system, Applied Biosystems/MDS SCIEX, Foster City, CA, USA). The LC separation was accomplished using a C30 carotenoid column YMC, 250 × 4.6 mm × 5 µm (Wilmington, NC) using mobile phases consisting of solvent A (MeOH/H_2_O (95:5, *v/v*) containing NH_4_HCO_2_ (5 mM)) and solvent B (MTBE/MeOH/H_2_O (90:7:3, *v/v/v*) containing NH_4_HCO_2_ (5 mM)) following the gradient elution starting from 0% B to 100% B in 45 min with a constant flow rate (1 mL/min) at 20 °C. Then, 20 mL of KP-BG leaf extract was injected into the YMC column using an autosampler. The optimized values of multiple reaction monitoring are listed in Table S1. Three independent extracts obtained from in vitro or ex vitro KP-BG leaves were analyzed in triplicate.

#### Comparative High-Performance Liquid Chromatography (HPLC) Analysis of Carotenoids in Carrot, KP-BG, and Lettuce

Extraction and analysis of carotenoids were performed following the protocols of Kim et al. [37] and Park et al. [39], respectively. The identification of (all-E)-α-carotene was performed by comparison of the RT with standard (all-E)-α-carotene) and absorption spectra were recorded by a diode array detector (DAD).

### 2.4. Composition of Fatty Acids

Lipids from KP-BG leaves were extracted following our optimized protocols [40,41], with minor modifications, originally based on a previous report [42]. First, 1 g of fresh leaves of KP-BG was mined with 20 mL of Ipa/Cyhex (10:12, *v/v*) and 0.075% Bht (*w/v*), sonicated for 10 min (JAC-2010; 300 W, 60 Hz), centrifuged (8000× *g* for 10 min at 4 °C), and the supernatant was collected. The sample was re-extracted with 20 mL of fresh solvent. Combined supernatants (approximately 40 mL) were partitioned with an equal volume of 1 M NaCl. The Ipa layer was collected, filtered through Na_2_SO_4_, and evaporated at 35 °C using a Büchi RE 111 rotary vacuum evaporator; the residue was dissolved in 3 mL of Cyhex/Dcm (1:3, *v/v*) containing 0.1% Bht, and stored at −20 °C.

Fatty acid methyl esters (FAMEs) were prepared using a BF3–MeOH solution (14% in methanol), according to the manufacturer’s guidelines, with minor modifications. First, 1 mL of KP-BG lipid sample was placed in a 5 mL glass vial and evaporated at 35 °C using a Büchi RE 111 rotary vacuum evaporator; 1 mL of BF3-MeOH solution was added, and then it was heated at 60 °C for 10 min. After cooling, the KP-BGFAME solution was washed with 1 M NaCl, recovered in 2 mL of Hex containing a small amount of Na_2_SO_4_, and filtered through a 0.45 µm nylon syringe filter into a vial.

FAMEs were quantitatively analyzed according to Saini et al. [41] using an Agilent (Agilent Technologies Canada, Inc.) 7890 B GC equipped with a flame ionization detector (FID), an autoinjector, and an SP-2560 100 m × 0.25 mm I.D. × 0.20 µm film thickness capillary GC column (Merck KGaA, Darmstadt, Germany). The column oven temperature was set to hold at 140 °C for 5 min, increased to 240 °C at a rate of 4 °C/min, and finally held at 240 °C for 15 min. Nitrogen was used as the carrier gas (2 mL/min). For GC–MS analysis, a QP2010 SE system (Shimadzu, Japan) was used. Three independent extracts (FAMEs) obtained from in vitro or ex vitro KP-BG leaves were analyzed in triplicate.

## 3. Results

### 3.1. Lipophilic Metabolite Content

The amounts of lipophilic metabolites in the KP-BG leaves are listed in Table 1. The lipophilic metabolites identified and measured in KP-BG leaves by LC–MRM–MS can be categorized as carotenoids, vitamin K1, tocopherols, and phytosterols. Higher amounts of total carotenoids (93.84 µg/g FW) and total tocopherols (46.22 µg/g FW) were found in the leaves of ex-vitro-raised KP-BG, whereas the levels of vitamin K1 (7.31 µg/g FW) and total phytosterols (44.40 µg/g FW) were higher in the leaves of in-vitro-grown KP-BG than ex-vitro-raised KP-BG.

**Figure 1:**
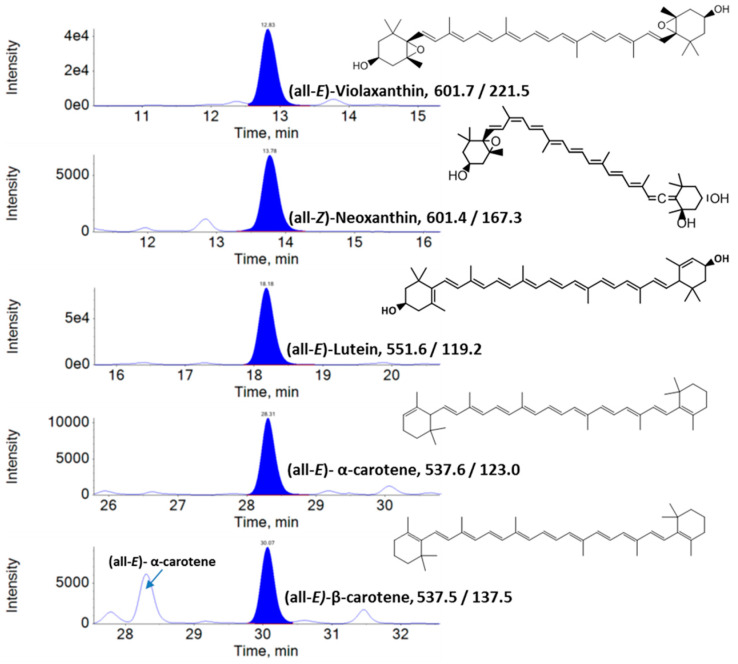
LC–MRM–MS chromatograms of carotenoids of KP-BG leaf extracts.

**Figure 2:**
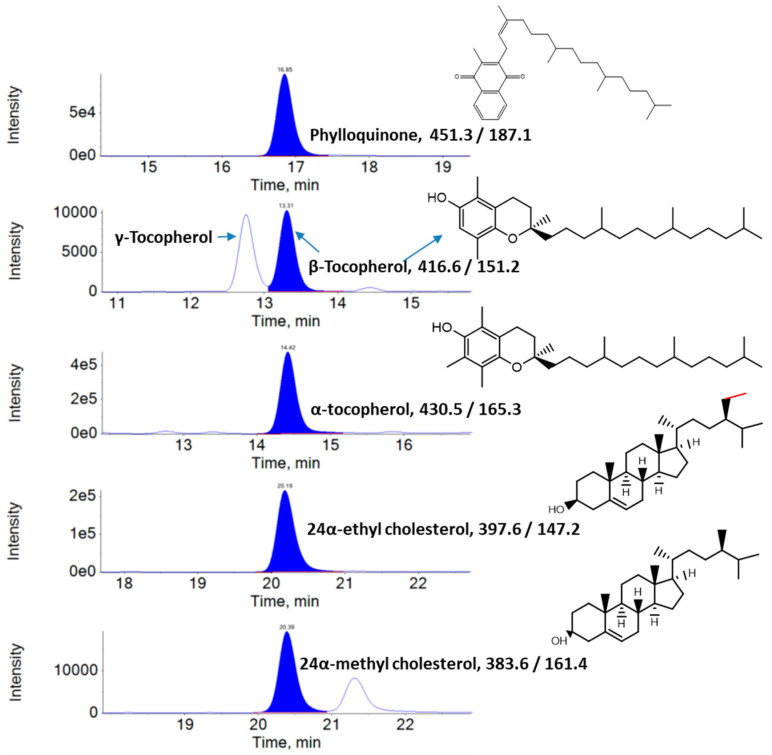
LC–MRM–MS chromatograms of phylloquinone (Vitamin K1), tocopherols, and phytosterols of KP-BG leaf extracts.

LC–MRM–MS chromatograms of carotenoids found in KP-BG leaf extracts are shown in Figure 1. Five carotenoids were identified in the KP-BG leaves. Among them, Lut (39.42–44.38 µg/g FW) was the major carotenoid, followed by α-Car (10.85–14.79 µg/g FW), Neo (10.10–12.30 µg/g FW), β-Car (10.61–11.33 µg/g FW), and Vio (9.42–11.03 µg/g FW) (Table 1). LC–MRM–MS chromatograms of vitamin K1, tocopherols, and phytosterols in KP-BG leaf extracts are shown in Figure 2. α-Toc (31.08–39.07 µg/g FW) was the predominant tocopherol in KP-BG leaves, followed by β-Toc (3.44–5.66 µg/g FW) and γ-Toc (3.08–5.49 µg/g FW). Two phytosterols were identified in the leaves of KP-BG. Specifically, 24α-ethyl cholesterol (30.55–36.62 µg/g FW) was the dominant phytosterol, followed by 24α-methyl cholesterol (7.14–7.78 µg/g FW).

### 3.2. Composition of Fatty Acids

**Figure 3:**
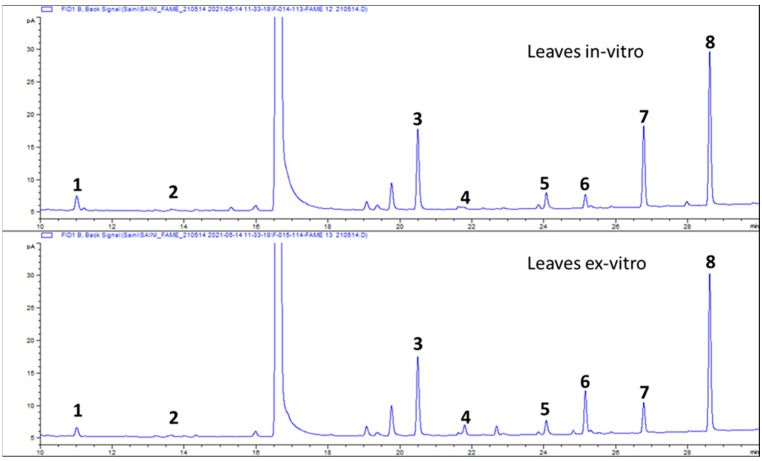
GC–FID chromatograms of FAMEs of KP-BG leaves. (1) C10:0 (Capric), (2) C12:0 (Lauric), (3) C16:0 (Palmitic), (4) C16:1 (Palmitoleic), (5) C18:0 (Stearic), (6) C18:1n9c (Oleic), (7) C18:2n6c (Linoleic), (8) C18:3n3 (α-Linolenic).

The GC chromatograms of FAMEs of KP-BG leaves are shown in Figure 3. Eight fatty acids were identified in the KP-BG leaves, as shown in Table 2. α-Linolenic (43.12%), palmitic (23.78%), oleic (12.28%), palmitoleic (3.64%), and lauric (1.14%) acids were the major fatty acids in the ex-vitro-raised KP-BG leaves, whereas linoleic (21.35%) and capric (5.47%) were the dominant fatty acids in the leaves of in-vitro-raised KP-BG. However, the stearic acid content (4.85–4.86%) in both leaf tissues was almost similar (Table 2). Higher levels of total saturated fatty acids (SFAs, 33.89%) and total polyunsaturated fatty acids (PUFAs, 61.03%) were found in KP-BG leaves grown in vitro. Ex-vitro-raised KP-BG leaves had a higher content of total monounsaturated fatty acids (MUFAs, 15.91%) than the KP-BG leaves grown in vitro (5.08%). The ratios of PUFAs:SFAs (1.80) and PUFAs:MUFAs (12.00) in the KP-BG leaves grown in vitro were higher than those in the ex-vitro-raised KP-BG leaves.

## 4. Discussion

In recent decades, there has been a growing interest in using byproducts (wastes) as a potential source for obtaining industrially important compounds. Ginger leaves have been reported to contain several bioactive metabolites [22,23,24,25,26,27]; however, the profile and content of lipophilic metabolites in KP-BG leaves have not been examined. Lipophilic compounds such as carotenoids, tocopherols, plant sterols, and fatty acids are important antioxidants that are used in the cosmetics, foods, and nutraceutical industries [32,43]. Plants and several microorganisms produce carotenoids as fat-soluble colored pigments, which are used as natural colorants in various industries [36,44]. These colored pigments are important components required for light harvesting and photoprotection in plants [28]. The profile and content of carotenoids in reproductive (flower, fruit, and seed) and vegetative (leaf and stem) organs vary with plant species, cultivar, growth stage, and environment. In this study, the ex-vitro-raised KP-BG leaves had higher total carotenoid content (93.84 µg/g FW) than the in-vitro-grown KP-BG leaves (80.40 µg/g FW). A similar result was observed in *Aronia melanocarpa* [45] and *Sedum dasyphyllum* [46]. Of the identified carotenoids, Lut was found to be a major component (44.38 µg/g FW) in KP-BG leaves (Table 1). Lut has been identified as the predominant carotenoid in leafy green vegetables and the mature leaves of many medicinal plants [37,39,45,46,47].

**Figure 4:**
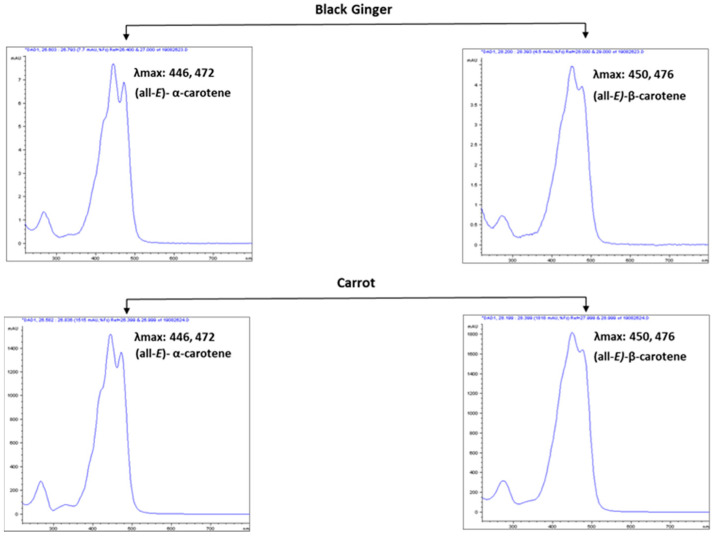
Absorption spectra recorded by diode array detector (DAD).

**Figure 5:**
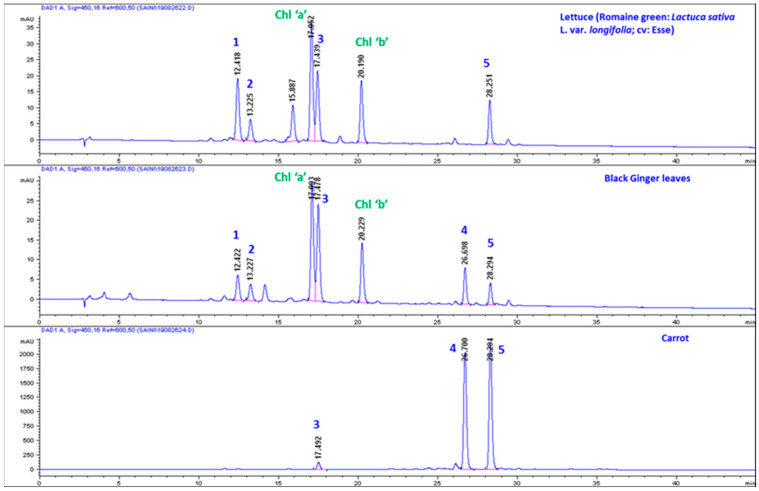
HPLC analysis of pigments in black ginger, carrot, and lettuce. (1) (all-E)-Violaxanthin; (2) (all-Z)-Neoxanthin; (3) (all-E)-Lutein; (4) (all-E)- α-carotene; (5) (all-E)-β-carotene; Chl: chlorophyll.

α-Car and β-Car are important provitamin A carotenoids found in *Cucurbita pepo*, *Daucus carota*, *Ipomoea batatas,* and some cultivars of apricot, beans, and squash at high concentrations [48,49]. However, these provitamin A carotenoids were found in green leaves at low concentrations. In addition, the β-Car content in leaf tissues is often higher than that α-Car [50]. In this study, the presence of a high amount of α-Car (similar to β-Car) in ginger foliage was surprising. Generally, in green foliage, complete conversion of α-Car to Lut causes the dominance of lutein and the absence of α-Car [51]. α-Car was absent in the leafy vegetables studied by Lakshminarayana et al. [51], including *Basella rubra*, *Peucedanum sowa*, *Moringa oleifera*, *Trigonella foenum-graecum*, *Spinacia oleracea*, *Sesbania grandiflora*, and *Raphanus sativus*. Similarly, α-Car was not recorded in the fresh-cut foliage of Romaine (red) (*Lactuca sativa* var. *romana*), Komatsuna (*Brassica rapa*var. *perviridis*), salad rocket (Garden rocket, Arugula) (*Eruca sativa*, syn. *E. vesicaria* subsp. *sativa*), wild rocket (perennial wall rocket) (*Diplotaxis tenuifolia*), and Batavian lettuce (*Lactuca sativa* L. var. *acephala*) [52]. Thus, considering the previous findings of the absence of α-Car in several green foliages and confirming its significant presence in ginger leaves, we recorded the absorbance spectrum by online HPLC–DAD, which was in addition to the confirmation by retention time and molecular mass of qualifier (Ql) and quantifier (Qt) transition ions of α-Car and other metabolites quantified by LC–MRM–MS. However, the MRM transition of m/z 537.5/137.5 is common between α-Car and β-Car. The retention time (28.31 and 30.07 min for α- and β-Car, respectively; Figure 1), transition of m/z 537.6/123.0 (produced selectively from α-carotene; Figure 1), and the absorbance spectrum can easily distinguish between α-Car (λmax: 446, 472) and β-Car (λmax: 450, 476) (Figure 4). Similarly, the chromatograms of lettuce and carrot were compared. Orange carrots are the most significant source of α-Car [53]. The α-Car peak was absent in lettuce, whereas it was dominant in ginger leaves and carrots (Figure 5).

Phylloquinone, another important antioxidant, is a lipid-soluble vitamin found in plants [54]. The phylloquinone content in KP-BG leaves (7.31 µg/g FW) (Table 1) was higher than that found in artichokes (16.2 µg·100 g^−1^ FW), broccoli (102 µg·100 g^−1^ FW), broccoli raab (242 µg·100 g^−1^ FW), carrot (8.3 µg·100 g^−1^ FW), celery (29.0 µg·100 g^−1^ FW), cucumber (16.4 µg·100 g^−1^ FW), lettuce ‘Green leaf’ (127 µg·100 g^−1^ FW), pepper (green) (7.1 µg·100 g^−1^ FW), potato (red) (3.2 µg·100 g^−1^ FW), sweet potato (1.8 µg·100 g^−1^ FW), radish (1.4 µg·100 g^−1^ FW) [55], and broccoli byproducts (leaves) (24.3 µg/g FW) [56]. Tocopherols are important bioactive compounds mainly obtained from photosynthetic organisms [57]. The levels of individual tocopherols differed among KP-BG leaves. γ-Toc (5.49 µg/g FW) and β-Toc (5.66 µg/g FW) levels were higher in in vitro KP-BG leaves than in ex vitro KP-BG leaves (Table 1). However, the major tocopherol (α-Toc) content in ex vitro KP-BG leaves (39.70 µg/g FW) was higher than that in in vitro KP-BG leaves (31.08 µg/g FW). The variation in the tocopherol content of KP-BG leaves may be due to differences in the growing environment, plant physiological status, and nutrient availability. The production of metabolites through plant organ cultures also changes according to environmental and nutritional factors [39,45,46]. It is worth mentioning that the α-Toc content in ex vitro KP-BG leaves (39.70 µg/g FW) is higher than that of various medicinal plants, such as *Tanacetum vulgare* (2.2 µg/g dry weight (DW), *Silene vulgaris* (11.21 µg/g DW), *Urtica dioica* (16.52 µg/g DW), and *Rosa canina* L. cv. Plovdiv 1 (34.05 µg/g DW) [58]. Moreover, 24α-ethyl cholesterol is a valuable phytosterol found abundantly in plants and has been reported to have antimicrobial, antioxidant, anti-inflammatory, antidiabetic, anticancer, and immunomodulatory activities [59]. In this study, 24α-ethyl cholesterol was found to be the major phytosterol in KP-BG leaves.

**Figure 6:**
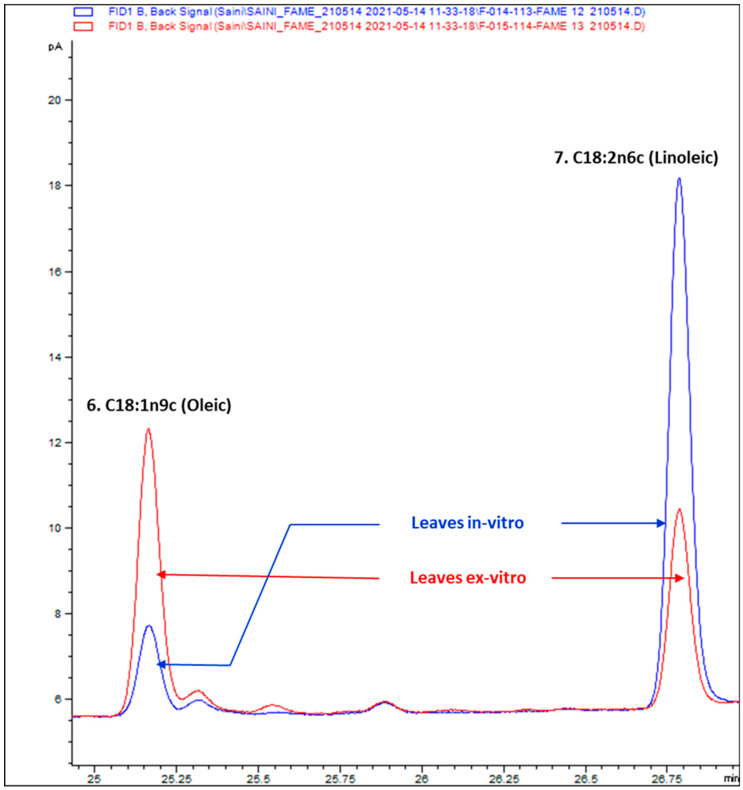
Comparison of oleic: linoleic acid peaks.

To the best of our knowledge, there are no reports on the fatty acid profile of KP-BG. In this study, the fatty acid composition of KP-BG leaves was examined using GC–FID/MS for the first time. α-Linolenic acid was found (39.68–43.12%) to be the most abundant fatty acid in KP-BG leaves (Table 2). Similarly, α-Linolenic acid was identified as the predominant fatty acid in the leaves of *Mertensia maritima* [39], *Aronia melanocarpa* [45], *Sedum dasyphyllum* [46], and *Ajuga multiflora* [60]. In the in-vitro-raised KP-BG leaves, the proportion of oleic acid (OA) decreased, and that of linoleic acid (LA) increased. A reverse trend was observed in the ex-vitro-grown KP-BG leaves (Table 2). In plants, ∆^12^-fatty acid desaturase catalyzes the conversion of C18:1n9c (OA) to C18:2n6c (LA) [61]. Comparison of OA:LA peaks can be discussed, as the OA:LA ratio is 0.17 in in-vitro-raised KP-BG leaves and 1.5 in leaves obtained from ex-vitro-grown KP-BG plants (Figure 6). OA has been reported to have anticancer activity and prevent cardiovascular diseases [62]. LA is an important PUFA that is essential for human life [63]. In this study, the KP-BG leaves raised in vitro had higher total PUFA content (61.03%) than the ex-vitro-grown KP-BG leaves (51.59%). In addition, the percentage of individual identified fatty acids varied among the KP-BG leaves. Similar results were also observed in in vitro cultures of *Sedum dasyphyllum* [46], *Ajuga multiflora* [60], *Argania spinosa* [64], and conifers [65]. It has been disclosed that plant growth regulators (PGR) could alter the fatty acid profile of treated plants [46,60]. The addition of PGR to in vitro culture media is often required to stimulate callus and shoot formation. Such calli and shoots (in-vitro-raised) may accumulate higher levels of fatty acids than ex-vitro-grown plants [46].

## 5. Conclusions

The lipophilic metabolite profiles and contents of KP-BG leaves were successfully analyzed using LC–MRM–MS and GC–FID/MS. The analysis and quantification of lipophilic components is an engaging task and, using the sophistication offered by the dual analytical approaches, we have been able to analyze and quantify the antioxidant compounds. The present report elaborately consolidates the bioactive components present in these leaves. In total, five carotenoids, three tocopherols, two phytosterols, eight fatty acids, and vitamin K were identified in KP-BG leaves. Further, the quantification data confirmed that Lut, α-Toc, 24α-ethyl cholesterol, and α-linolenic acid were the major lipophilic metabolites in KP-BG leaves. In addition, the content of α-car was higher than that of β-car. The findings of this study confirmed that KP-BG byproducts are a rich source of lipophilic antioxidants. However, the beneficial effects of the individual lipophilic compounds identified in KP-BG byproducts should be studied. This study confirms that KP-BG leaves are a rich reservoir of the numerous listed antioxidant-rich bioactive metabolites and emphasizes the need to exploit these resources for human benefit.

## Figures and Tables

**Table 1:** Contents of major lipophilic metabolites from the KP-BG leaves, quantified (µg/g FW) by the LC–MRM–MS method.

S/No	Analyte	In Vitro Leaves	Ex Vitro Leaves
1	(all-*E*)-Violaxanthin	9.42	11.03
2	(all-*Z*)-Neoxanthin	10.10	12.30
3	(all-*E*)-Lutein	39.42	44.38
4	(all-*E*)-α-carotene	10.85	14.79
5	(all-*E*)-β-carotene	10.61	11.33
6	Total carotenoids (S/No. 1+2+3+4+5)	80.40	93.84
7	Phylloquinone (Vitamin K1)	7.31	7.25
8	γ-Tocopherol	5.49	3.08
9	β-Tocopherol	5.66	3.44
10	α-Tocopherol	31.08	39.70
11	Total tocopherols (S/No. 8+9+10)	42.23	46.22
12	24α-ethyl cholesterol	36.62	30.55
13	24α-methyl cholesterol	7.78	7.14
14	Total phytosterols	44.40	37.69

**Table 2:** Composition of fatty acids in KP-BG leaves.

S/No	FAME	RT (Minute)	In Vitro Leaves	Ex Vitro Leaves
1	C10:0 (Capric)	11.029	5.47	3.03
2	C12:0 (Lauric)	13.666	1.03	1.14
3	C_16:0_ (Palmitic)	20.509	22.53	23.78
4	C_16:1_ (Palmitoleic)	21.744	1.37	3.64
5	C_18:0_ (Stearic)	24.082	4.86	4.85
6	C_18:1n9c_ (Oleic)	25.169	3.71	12.28
7	C_18:2n6c_ (Linoleic)	26.789	21.35	8.17
8	C_18:3n3_ (α-Linolenic)	28.625	39.68	43.12
	Oleic: linoleic acid		0.17	1.50
	Total SFAs		33.89	32.80
	Total MUFAs		5.08	15.91
	Total PUFAs		61.03	51.29
	PUFAs: SFAs		1.80	1.56
	PUFAs: MUFAs		12.00	3.22

Values are % of total fatty acids. SFAs: total saturated fatty acids; MUFAs: total monounsaturated fatty acids; PUFAs: total polyunsaturated fatty acids; RT: retention time.

## Data Availability

Data is contained within the article.

## Supplementary Materials

The following are available online at https://www.mdpi.com/article/10.3390/antiox10101573/s1, Table S1: Optimized values of declustering potential (DP), entrance potential (EP), collision energy (CE), cell exit potential (CXP) and retention time (Rt) of qualifier (Ql) and quantifier (Qt) transition used for the liquid chromatography (LC)-multiple reaction monitoring (MRM)-mass spectrometry (MS) quantification of major lipophilic metabolites from the *Kaempferia parviflora* (black ginger) (KP-BG) leaves.

## References

[1] Techaprasan J., Klinbunga S., Ngamriabsakul C., Jenjittikul T. (2010). Genetic variation of *Kaempferia* (Zingiberaceae) in Thailand based on chloroplast DNA (*psbA*-*trnH* and *petA*-*psbJ*) sequences. Genet. Mol. Res..

[2] Elshamy A.I., Mohamed T.A., Essa A.F., Abd-El Gawad A.M., Alqahtani A.S., Shahat A.A., Yoneyama T., Farrag A.R.H., Noji M., El-Seedi H.R. (2019). Recent advances in *Kaempferia* phytochemistry and biological activity: A comprehensive review. Nutrients.

[3] Kochuthressia K., Britto S.J., Jaseentha M., Raphael R. (2012). In vitro antimicrobial evaluation of *Kaempferia galanga* L. rhizome extract. Am. J. Biotechnol. Mol. Sci..

[4] Ridtitid W., Sae-Wong C., Reanmongkol W., Wongnawa M. (2008). Antinociceptive activity of the methanolic extract of *Kaempferia galanga* Linn. in experimental animals. J. Ethnopharmacol..

[5] Ahmed F.R.S., Amin R., Hasan I., Asaduzzaman A., Kabir S.R. (2017). Antitumor properties of a methyl-β-d-galactopyranoside specific lectin from *Kaempferia rotunda* against Ehrlich ascites carcinoma cells. Int. J. Biol. Macromol..

[6] Kobayashi S., Kato T., Azuma T., Kikuzaki H., Abe K. (2015). Anti-allergenic activity of polymethoxyflavones from *Kaempferia parviflora*. J. Funct. Foods.

[7] Hidaka M., Horikawa K., Akase T., Makihara H., Ogami T., Tomozawa H., Tsubata M., Ibuki A., Matsumoto Y. (2017). Efficacy of *Kaempferia parviflora* in a mouse model of obesity-induced dermatopathy. J. Nat. Med..

[8] Win N.N., Ngwe H., Abe I., Morita H. (2017). Naturally occurring Vpr inhibitors from medicinal plants of Myanmar. J. Nat. Med..

[9] Yeap Y.S.Y., Kassim N.K., Ng R.C., Ee G.C.L., Saiful Yazan L., Musa K.H. (2017). Antioxidant properties of ginger (*Kaempferia angustifolia* Rosc.) and its chemical markers. Int. J. Food Prop..

[10] Sawasdee P., Sabphon C., Sitthiwongwanit D., Kokpol U. (2009). Anticholinesterase activity of 7-methoxyflavones isolated from *Kaempferia parviflora*. Phytother. Res..

[11] Kaewkroek K., Wattanapiromsakul C., Matsuda H., Nakamura S., Tewtrakul S. (2017). Anti-inflammatory activity of compounds from *Kaempferia marginata* rhizomes. Songklanakarin J. Sci. Technol..

[12] Plaingam W., Sangsuthum S., Angkhasirisap W., Tencomnao T. (2017). *Kaempferia parviflora* rhizome extract and *Myristica fragrans* volatile oil increase the levels of monoamine neurotransmitters and impact the proteomic profiles in the rat hippocampus: Mechanistic insights into their neuroprotective effects. J. Tradit. Complement. Med..

[13] Shanbhag T.V., Sharma C., Adiga S., Bairy K., Shenoy S., Shenoy G. (2006). Wound healing activity of alcoholic extract of *Kaempferia galanga* in Wistar rats. Indian J. Physiol. Pharmacol..

[14] Pripdeevech P., Pitija K., Rujjanawate C., Pojanagaroon S., Kittakoop P., Wongpornchai S. (2012). Adaptogenic-active components from *Kaempferia parviflora* rhizomes. Food Chem..

[15] Azuma T., Kayano S.-I., Matsumura Y., Konishi Y., Tanaka Y., Kikuzaki H. (2011). Antimutagenic and glucosidase inhibitory effects of constituents from *Kaempferia parviflora*. Food Chem..

[16] Chaipech S., Morikawa T., Ninomiya K., Yoshikawa M., Pongpiriyadacha Y., Hayakawa T., Muraoka O. (2012). Structures of two new phenolic glycosides, kaempferiaosides A and B, and hepatoprotective constituents from the rhizomes of *Kaempferia parviflora*. Chem. Pharm. Bull..

[17] Thao N.P., Luyen B.T.T., Lee S.H., Jang H.D., Kim Y.H. (2016). Anti-osteoporotic and antioxidant activities by rhizomes of *Kaempferia parviflora* wall. Ex Baker. Nat. Prod. Sci..

[18] Tewtrakul S., Subhadhirasakul S., Karalai C., Ponglimanont C., Cheenpracha S. (2009). Anti-inflammatory effects of compounds from *Kaempferia parviflora* and *Boesenbergia pandurata*. Food Chem..

[19] Nakao K., Murata K., Deguchi T., Itoh K., Fujita T., Higashino M., Yoshioka Y., Matsumura S.-I., Tanaka R., Shinada T. (2011). Xanthine oxidase inhibitory activities and crystal structures of methoxyflavones from *Kaempferia parviflora* rhizome. Biol. Pharm. Bull..

[20] Sulaiman M.R., Zakaria Z.A., Duad I.A., Hidayat M.T. (2008). Antinociceptive and anti-inflammatory activities of the aqueous extract of *Kaempferia galanga* leaves in animal models. J. Nat. Med..

[21] Ali M.S., Dash P.R., Nasrin M. (2015). Study of sedative activity of different extracts of *Kaempferia galanga* in Swiss albino mice. BMC Complement. Altern. Med..

[22] Chan E.W.C., Lim Y.Y., Wong L.F., Lianto F.S., Wong S.K., Lim K.K., Joe C.E., Lim T.Y. (2008). Antioxidant and tyrosinase inhibition properties of leaves and rhizomes of ginger species. Food Chem..

[23] Park H.Y., Kim K.-S., Ak G., Zengin G., Cziáky Z., Jekő J., Adaikalam K., Song K., Kim D.H., Sivanesan I. (2021). Establishment of a rapid micropropagation system for *Kaempferia parviflora* wall. Ex Baker: Phytochemical analysis of leaf extracts and evaluation of biological activities. Plants.

[24] Preetha T.S., Hemanthakumar A.S., Krishnan P.N. (2016). A comprehensive review of *Kaempferia galanga* L. (Zingiberaceae): A high sought medicinal plant in tropical Asia. J. Med. Plants Stud..

[25] Chan E.W.C., Lim Y.Y., Wong S.K., Lim K.K., Tan S.P., Lianto F.S., Yong M.Y. (2009). Effects of different drying methods on the antioxidant properties of leaves and tea of ginger species. Food Chem..

[26] Bhuiyan M.N.I., Begum J., Anwar M.N. (2008). Essential oils of leaves and rhizomes of *Kaempferia galanga* Linn. Chittagong Univ. J. Biol. Sci..

[27] Norhayati Y., Nurulhidayah A., Rini Zunnurni M.J., Norliana A.R., Norliana W., Mohd Ifwat I. (2018). Antioxidative constituents of selected Malaysian ‘Ulam’. Sci. Int..

[28] Sandmann G. (2019). Antioxidant protection from UV- and light-stress related to carotenoid structures. Antioxidants.

[29] Lim G.H., Singhal R., Kachroo A., Kachroo P. (2017). Fatty acid- and lipid-mediated signaling in plant defense. Annu. Rev. Phytopathol..

[30] Cabianca A., Müller L., Pawlowski K., Dahlin P. (2021). Changes in the plant β-sitosterol/stigmasterol ratio caused by the plant parasitic nematode *Meloidogyne incognita*. Plants.

[31] Ma J., Qiu D., Pang Y., Gao H., Wang X., Qin Y. (2020). Diverse roles of tocopherols in response to abiotic and biotic stresses and strategies for genetic biofortification in plants. Mol. Breed..

[32] Chang K.H., Cheng M.L., Chiang M.C., Chen C.M. (2018). Lipophilic antioxidants in neurodegenerative diseases. Clin. Chim. Acta Int. J. Clin. Chem..

[33] Pogačnik L., Ota A., Ulrih N.P. (2020). An overview of crucial dietary substances and their modes of action for prevention of neurodegenerative diseases. Cells.

[34] Shin G.H., Kim J.T., Park H.J. (2015). Recent developments in nanoformulations of lipophilic functional foods. Trends Food Sci. Technol..

[35] Oppedisano F., Macrì R., Gliozzi M., Musolino V., Carresi C., Maiuolo J., Bosco F., Nucera S., Zito M.C., Guarnieri L. (2020). The anti-inflammatory and antioxidant properties of n-3 PUFAs: Their role in cardiovascular protection. Biomedicines.

[36] Saini R.K., Moon S.H., Gansukh E., Keum Y.S. (2018). An efficient one-step scheme for the purification of major xanthophyll carotenoids from lettuce, and assessment of their comparative anticancer potential. Food Chem..

[37] Kim D.E., Shang X., Assefa A.D., Keum Y.S., Saini R.K. (2018). Metabolite profiling of green, green/red, and red lettuce cultivars: Variation in health beneficial compounds and antioxidant potential. Food Res. Int..

[38] Kim D.H., Enkhtaivan G., Saini R.K., Keum Y.S., Kang K.W., Sivanesan I. (2019). Production of bioactive compounds in cladode culture of *Turbinicarpus valdezianus* (H. Moeller) Glass & R. C. Foster. Ind. Crop. Prod..

[39] Park H.Y., Kim D.H., Saini R.K., Gopal J., Keum Y.S., Sivanesan I. (2019). Micropropagation and quantification of bioactive compounds in *Mertensia maritima* (L.) gray. Int. J. Mol. Sci..

[40] Saini R.K., Assefa A.D., Keum Y.-S. (2021). Spices in the Apiaceae family represent the healthiest fatty acid profile: A aystematic comparison of 34 widely used spices and herbs. Foods.

[41] Saini R.K., Rauf A., Khalil A.A., Ko E.-Y., Keum Y.-S., Anwar S., Alamri A., Rengasamy K.R.R. (2021). Edible mushrooms show significant differences in sterols and fatty acid compositions. S. Afr. J. Bot..

[42] Cruz R., Casal S., Mendes E., Costa A., Santos C., Morais S. (2012). Validation of a single-extraction procedure for sequential analysis of vitamin E, cholesterol, fatty acids, and total fat in seafood. Food Anal. Methods.

[43] Dima Ş., Dima C., Iordăchescu G. (2015). Encapsulation of functional lipophilic food and drug biocomponents. Food Eng. Rev..

[44] Langi P., Kiokias S., Varzakas T., Proestos C. (2018). Carotenoids: From plants to food and feed industries. Methods Mol. Biol. Clifton NJ.

[45] Sivanesan I., Saini R.K., Kim D.H. (2016). Bioactive compounds in hyperhydric and normal micropropagated shoots of *Aronia melanocarpa* (michx.) Elliott. Ind. Crops Prod..

[46] Park H.Y., Saini R.K., Gopal J., Keum Y.-S., Kim D.H., Lee O., Sivanesan I. (2017). Micropropagation and subsequent enrichment of carotenoids, fatty acids, and tocopherol contents in *Sedum dasyphyllum* L.. Front. Chem..

[47] Yoon G.-A., Yeum K.-J., Cho Y.-S., Chen C.-Y.O., Tang G., Blumberg J.B., Russell R.M., Yoon S., Lee-Kim Y.C. (2012). Carotenoids and total phenolic contents in plant foods commonly consumed in Korea. Nutr. Res. Pr..

[48] Saini R.K., Nile S.H., Park S.W. (2015). Carotenoids from fruits and vegetables: Chemistry, analysis, occurrence, bioavailability and biological activities. Food Res. Int..

[49] Pan Z., Sun Y., Zhang F., Guo X., Liao Z. (2019). Effect of thermal processing on carotenoids and folate changes in six varieties of sweet potato (*Ipomoes batata* L.). Foods.

[50] Ponder A., Hallmann E. (2019). Phenolics and carotenoid contents in the leaves of different organic and conventional raspberry (*Rubus idaeus* L.) cultivars and their in vitro activity. Antioxidants.

[51] Lakshminarayana R., Raju M., Krishnakantha T.P., Baskaran V. (2005). Determination of major carotenoids in a few Indian leafy vegetables by high-performance liquid chromatography. J. Agric. Food Chem..

[52] Saini R.K., Shang X.M., Ko E.Y., Choi J.H., Kim D., Keum Y.-S. (2016). Characterization of nutritionally important phytoconstituents in minimally processed ready-to-eat baby-leaf vegetables using HPLC–DAD and GC–MS. J. Food Meas. Charact..

[53] Purkiewicz A., Ciborska J., Tańska M., Narwojsz A., Starowicz M., Przybyłowicz K.E., Sawicki T. (2020). The impact of the method extraction and different carrot variety on the carotenoid profile, total phenolic content and antioxidant properties of juices. Plants.

[54] Basset G.J., Latimer S., Fatihi A., Soubeyrand E., Block A. (2017). Phylloquinone (vitamin K1): Occurrence, biosynthesis and functions. Mini Rev. Med. Chem..

[55] Damon M., Zhang N.Z., Haytowitz D.B., Booth S.L. (2005). Phylloquinone (vitamin K1) content of vegetables. J. Food Compos. Anal..

[56] Liu M., Zhang L., Ser S., Cumming J., Ku K. (2018). Comparative phytonutrient analysis of broccoli byproducts: The potentials for broccoli byproduct utilization. Molecules.

[57] Szewczyk K., Chojnacka A., Górnicka M. (2021). Tocopherols and tocotrienols—Bioactive dietary compounds; what is certain, what is doubt?. Int. J. Mol. Sci..

[58] Mihaylova D., Vrancheva R., Petkova N., Ognyanov M., Desseva I., Ivanov I., Popova M., Popova A. (2018). Carotenoids, tocopherols, organic acids, charbohydrate and mineral content in different medicinal plant extracts. Z. Nat. C. J. Biosci..

[59] Babu S., Jayaraman S. (2020). An update on β-sitosterol: A potential herbal nutraceutical for diabetic management. Biomed. Pharmacother..

[60] Sivanesan I., Saini R.K., Noorzai R., Zamany A.J., Kim D.H. (2016). In vitro propagation, carotenoid, fatty acid and tocopherol content of *Ajuga multiflora* Bunge. 3 Biotech.

[61] Chen P.-Y., Hsieh M.-J., Tsai Y.-T., Chung H.-H., Shyur L.-F., Hsieh C.-H., To K.-Y. (2020). Transformation and characterization of Δ12-fatty acid acetylenase and Δ12-oleate desaturase potentially involved in the polyacetylene biosynthetic pathway from *Bidens pilosa*. Plants.

[62] Sales-Campos H., Reis de Souza P., Crema Peghini B., Santana da Silva J., Ribeiro Cardoso C. (2013). An overview of the modulatory effects of oleic acid in health and disease. Mini Rev. Med. Chem..

[63] Marangoni F., Agostoni C., Borghi C., Catapano A.L., Cena H., Ghiselli A., La Vecchia C., Lercker G., Manzato E., Pirillo A. (2020). Dietary linoleic acid and human health: Focus on cardiovascular and cardiometabolic effects. Atherosclerosis.

[64] Koufan M., Belkoura I., Mazri M.A., Amarraque A., Essatte A., Elhorri H., Zaddoug F., Alaoui T. (2020). Determination of antioxidant activity, total phenolics and fatty acids in essential oils and other extracts from callus culture, seeds and leaves of *Argania spinosa* (L.) Skeels. Plant Cell Tissue Organ Cult..

[65] Semenova N.V., Makarenko S.P., Shmakov V.N., Konstantinov Y.M., Dudareva L.V. (2017). Fatty acid composition of total lipids from needles and cultured calluses of conifers *Pinus sylvestris* L., *Picea pungens* Engelm., *Pinus koraiensis* Siebold & Zucc., and *Larix sibirica* Ledeb. Biochem. Suppl. Ser. A Membr. Cell Biol..

