# Supplementary material for: Analysis of Lipophilic Antioxidants in the Leaves of Kaempferia parviflora Wall. Ex Baker Using LC–MRM–MS and GC–FID/MS
Source: Antioxidants (Basel). 2021 Oct 5;10(10):1573. doi: 10.3390/antiox10101573 (PMC8533615; doi:10.3390/antiox10101573)
Supplement: Supplementary file 1 [file antioxidants-10-01573-s001.zip › antioxidants-1381802-supplementary.pdf]

Analysis of lipophilic antioxidants in the leaves of *Kaempferia parviflora* Wall. Ex Baker using LC-MRM-MS and GC-FID/MS

Table S1. Optimized values of declustering potential (DP), entrance potential (EP), collision energy (CE), cell exit potential (CXP) and retention time (Rt) of qualifier (Ql) and quantifier (Qt) transition used for the liquid chromatography (LC)-multiple reaction monitoring (MRM)-mass spectrometry (MS) quantification of major lipophilic metabolites from the *Kaempferia parviflora* (black ginger) (KP-BG) leaves.

Analyte	Q1 Mass	Q3 Mass	RT (Min)	DP (V)	EP (V)	CE (eV)	Transition
Violaxanthin (1)	601.7	221.5	12.83	70	10	40	Qt
Violaxanthin (2)	601.6	165.4	12.83	70	10	50	Ql
Neoxanthin (1)	601.4	167.3	13.78	50	10	30	Qt
Neoxanthin (2)	601.7	167.3	13.78	50	10	30	Ql
Lutein (1)	551.6	119.2	18.18	70	10	50	Qt
Lutein (2)	551.6	133.2	18.18	70	10	50	Ql
α-Carotene (1)	537.6	123.0	28.31	50	10	40	Qt
α-Carotene (2)	537.6	95.1	28.31	50	10	40	Ql
β-Carotene (1)	537.5	137.5	30.07	50	10	40	Qt
β-Carotene (2)	537.5	177.5	30.07	50	10	40	Ql
Phylloquinone (1)	451.3	187.1	16.85	50	10	30	Qt
Phylloquinone (2)	451.0	197.0	16.85	50	10	30	Ql
γ-Tocopherol	416.6	151.2	12.73	65	10	50	Qt/Ql
β-Tocopherol	416.6	151.2	13.31	65	10	50	Qt/Ql
α-Tocopherol (1)	430.5	165.3	14.42	65	10	40	Qt
α-Tocopherol (2)	430.6	205.3	14.42	65	10	40	Ql
24α-ethyl cholesterol (1)	397.6	147.2	20.19	60	10	40	Qt
24α-ethyl cholesterol (2)	397.5	159.2	20.19	60	10	40	Ql
24α-ethyl cholesterol (3)	397.6	161.4	20.19	60	10	40	Ql
24α-methyl cholesterol (1)	383.6	161.4	20.39	70	10	40	Ql
24α-methyl cholesterol (2)	383.6	147.3	20.39	70	10	40	Qt
24α-methyl cholesterol (3)	383.6	135.3	20.39	70	10	40	Ql
trans-β-apo-8'-carotenal (1)	417.6	209.5	21.54	50	10	40	Qt
trans-β-apo-8'-carotenal (2)	417.6	145.9	21.56	50	10	40	Ql
trans-β-apo-8'-carotenal (3)	417.6	119.4	21.55	50	10	40	Ql

5-α-Cholestan-3-β-ol (1)	371.7	109.3	22.09	70	10	40	Qt
5-α-Cholestan-3-β-ol (2)	371.7	135.3	22.08	70	10	40	Ql
5-α-Cholestan-3-β-ol (3)	371.7	149.4	22.08	70	10	40	Ql
